# Mid-Term Clinical and Radiographic Results of Complex Hip Revision Arthroplasty Based on 3D Life-Sized Model: A Prospective Case Series

**DOI:** 10.3390/jcm13185496

**Published:** 2024-09-17

**Authors:** Francesco La Camera, Vincenzo Di Matteo, Alessandro Pisano, Edoardo Guazzoni, Carlo Maria Favazzi, Katia Chiappetta, Emanuela Morenghi, Guido Grappiolo, Mattia Loppini

**Affiliations:** 1 IRCCS Humanitas Research Hospital, 20089 Milan, Italy; francesco.lacamera@humanitas.it (F.L.C.); e.guazzoni@gmail.com (E.G.); favazzi.c@gmail.com (C.M.F.); katia.chiappetta@humanitas.it (K.C.); biostatistica.medica.pubblicazioni@outlook.it (E.M.); guido.grappiolo@mac.com (G.G.); mattia.loppini@hunimed.eu (M.L.); 2 Fondazione Livio Sciutto Onlus, Campus Savona, Università degli Studi di Genova, 17100 Savona, Italy; 3 Department of Biomedical Sciences, Humanitas University, 20090 Milan, Italy; alepisano08@gmail.com; 4 Orthopedics and Trauma Surgery Unit, Department of Aging, Orthopedic and Rheumatologic Sciences, Fondazione Policlinico Universitario Agostino Gemelli IRCCS, 00168 Rome, Italy

**Keywords:** complex acetabular reconstruction, pre-operative planning, revision total hip arthroplasties, 3D printed models

## Abstract

**Background:** The pre-operative three-dimensional (3D) assessment of acetabular bone defects may not be evaluated properly with conventional radiographic and computed tomography images. This paper reports mid-term clinical and radiographic outcomes of complex revision total hip arthroplasty (r-THA) based on a 3D life-sized printed model. **Methods:** Patients who underwent r-THA for septic or aseptic acetabular loosening with acetabular defects Paprosky types IIc, IIIa, and IIIb between 2019 and 2021 were included. The outcomes of the study were to determine clinical and radiographic assessment outcomes at the time of the last follow-up. **Results:** 25 patients with mean age of 62.9 ± 10.8 (18–83) years old were included. The mean Harris hip score improved from 34.8 ± 8.1 pre-operative to 81.6 ± 10.4 points (*p* < 0.001). The mean visual analog scale decreased from 6.7 ± 1.4 points pre-operative to 2.4 ± 1.0 points (*p* < 0.001). The mean limb length discrepancy improved from −2.0 ± 1.2 cm pre-operative to −0.6 ± 0.6 cm (*p* < 0.001). The mean vertical position of the center of rotation (COR) changed from 3.5 ± 1.7 cm pre-operative to 2.0 ± 0.7 cm (*p* < 0.05). The mean horizontal COR changed from 3.9 ± 1.5 cm pre-operative to 3.2 ± 0.5 cm (*p* < 0.05). The mean acetabular component abduction angle changed from 59.7° ± 29.6° pre-operative to 46° ± 3.9 (*p* < 0.05). **Conclusions:** A three-dimensional-printed model provides an effective connection between the pre-operative bone defects’ evaluation and the intraoperative findings, enabling surgeons to select optimal surgical strategies.

## 1. Introduction

In recent years, the number of total hip arthroplasty (THA) procedures performed worldwide has increased, with the greatest increase in patients aged between 45 and 54 years old [1]. The number of revision total hip arthroplasties due to various complications, especially when performed in younger patients, also increased accordingly [2,3]. During acetabular revision procedures, massive osteolysis with Paprosky types IIc, IIIa, and IIIb acetabular defects have been reported. A stable fixation of the acetabular component is very challenging due to bone loss, necessitating structural bone grafting, metal augments, and even custom prosthetic implants. Careful pre-operative planning is crucial for the proper reconstruction of hip biomechanics. A comprehensive three-dimensional (3D) understanding of the abnormal bony anatomy is required to accurately evaluate and manage acetabular bone defects. Preoperative acetabular revision procedure planning is performed on traditional X-ray and computed tomography (CT) images, leaving the surgeon to conceptualize two-dimensional (2D) reconstructions into 3D. An accurate evaluation of the residual bone stock and 3D extension of the acetabular bone defects on 2D images is very challenging, and the surgeon’s experience has a high impact. The evaluation of periprosthetic bone loss and fracture lines is more accurate on a CT scan, but metal hardware in the hip usually causes significant scattering of the images. In addition, CT evaluation on a 2D screen still poses some difficulty in acetabular anatomy evaluation, increasing difficulty in surgical planning, and the surgeon is frequently forced to change the type of prosthetic implant during surgery or increase the duration of surgery, but intraoperative blood loss and related complications can also result.

Three-dimensional printing is a process of design and manufacturing, in a layer-by-layer construction, of anatomically detailed models. Bucking et al. [4] showed a typical process workflow for manufacturing medical models starting from patient anatomy captured via medical imaging, such as computed tomography (CT), followed by constructing a 3D model geometry for augmenting manufacturing using segmentation algorithms. In the last few years, the application in the orthopedic field has increased, arising from the ability of 3D printed anatomical model products to be manufactured to a high degree of accuracy using biocompatible materials [5].

In surgical applications, 3D models of patient-specific (PS) anatomical structures are used to create a model which gives a better understanding of the complex pathology and anatomy of the patient, finding great use in pre-operative planning, surgical guides, personalized implants, intraoperative prosthetic positioning, and customized prostheses [6,7]. The application of 3D-printed models during r-THA allows the surgeon to perform accurate reconstruction of the center of rotation (COR) and hip biomechanics. The restoration of the COR balances the stress distribution, reduces the wear of the polyethylene inserts, and prolongs prosthetic implant survival [8]. Regarding hip pathology, currently the major fields of application of 3D printing planning are: treatment of proximal femoral fractures [9], treatment of acetabular fractures [10], hip arthroplasties [11], and reconstructive oncological surgery [12]. According to the current literature, 3D-printed models facilitate surgical planning, fracture reduction, soft tissue trauma, duration of surgery, and bone and blood loss. This prospective cohort study describes a single cohort of patients with severe acetabular bone defects undergoing acetabular revision, based on a 3D life-sized model in a high-volume single center; all surgeries were performed by a single surgeon highly experienced in joint replacement surgery. The aim of the study was to determine clinical and radiographic assessment outcomes at the time of the last follow-up.

## 2. Materials and Methods

### 2.1. Protocol

**Figure 1:**
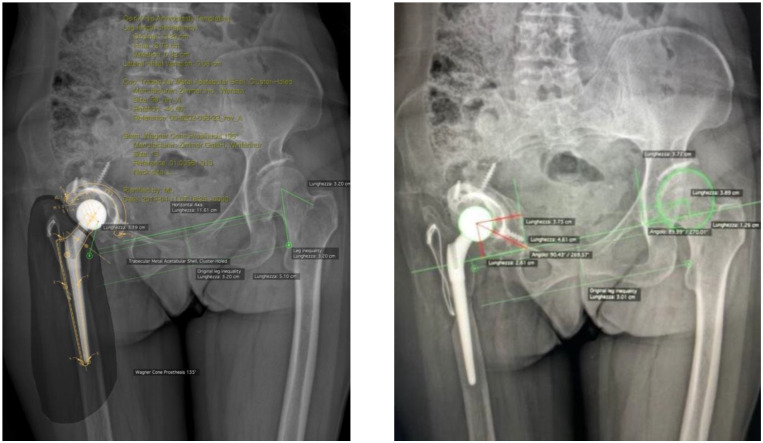
Pre-operative radiograph (AP view) shows AP pelvis in a 52 year-old woman with aseptic loosening of the acetabular cup with Paprosky type IIIa acetabular defect. The radiograph demonstrates supero medial migration of the hip COR (26 mm above the interdrop line), absence of the Kohler’s line, and ischial osteolysis. The two lines positioned at the tip of the lesser trochanter of each femur are used to measure the limb length discrepancy. In the present case, the pre-operative limb length discrepancy was 32.0 mm.

The present prospective cohort study included all patients who underwent r-THA surgery following septic or aseptic acetabular loosening, performed by senior surgeons experienced in join revision arthroplasties, from April 2019 to December 2021 at the IRCCS Humanitas Research Hospital, Italy. Patients were identified from hospital medical records, and the eligibility criteria included patients with acetabular bone defects Paprosky types IIc, IIIa, and IIIb who sustained acetabular revision arthroplasty based on a 3D life-sized model. Participants’ exclusion criteria were acetabular bone defects Paprosky types I, IIa, and IIb, pelvic discontinuity, recurrent dislocations, periprosthetic fractures, hip implant malposition, and patients under 18 years old. All included patients will be followed for a clinical and radiological follow-up in 2 years at least. All individual participants signed a written informed consent form for surgery and a written informed consent form to be included in the registry of orthopedic surgical procedures within the scope of research and improvement of clinical practice. All surgeries were performed with a post-erolateral approach by a single highly experienced surgeon. The demographic characteristics of the participants, including gender, age, BMI, diagnosis, details of the operative strategy treatments employed, r-THA or acetabular revision alone, type and diameter of cup implanted, augment, insert, bone graft, cage, bioball, number of previous surgeries, duration time (minutes), blood loss (ml), length of hospital stay (LOS), and post-operative complications, were collected from medical file records. A three-dimensional-printed model of the femur and pelvis based on a CT scan was developed for the enrolled patient. Clinical and radiographic evaluations were performed pre-operatively, immediately after surgery and subsequently at 1 month, 3 months, 6 months, 1 year, 2 years, and 5 years as standard protocol. Functional recovery was determined by an orthopedic surgeon by the comparing pre-operative and post-operative physical examinations with the Harris Hip Score (HHS) measurements, the visual analogue scale (VAS) for pain, and the presence of limping (none, low, moderate, and severe). A range of motion (ROM) evaluation was performed with a universal goniometer according to standard guidelines [13,14]. All patients had conventional anteroposterior (AP) radiographs of the pelvis and false profile (FP) projection, with patients in standing position pre-operatively and post-operatively during the follow-up evaluations. The pre-operative acetabular bone defects were classified according to the Paprosky classification on pre-operative AP radiographs using four bone landmarks: radiological tear defect, Kohler’s line integrity, ischial lysis, and superior defect with cranial migration of COR of the hip [15]. A defects classification was performed by two independent testers—a radiologist experienced in musculoskeletal radiology and a senior orthopedic surgeon who was not involved in the surgery; all disagreements were resolved using the intraoperative description of the defects reported in the clinical records. The limb length discrepancy (LLD) was assessed pre-operatively and post-operatively on digital radiographs in AP view with Hip Arthroplasty Templating 2.4.3 software running with 64-bit OsiriX v.5.8.1. The LLD measurement was performed using the ischial tuberosities and the lesser trochanter as reference points. The correct COR of the hip undergoing r-THA was identified considering the contralateral hip COR if it was not affected by arthritis, dysplasia, or other hip diseases. When the contralateral COR was not identifiable, we employed the methodology described by Ranawat et al. to identify the native COR and the superior and inferior border of the acetabulum [16]. Pre-operative and post-operative COR were also measured on the vertical axis of the radiological tear and on the horizontal axis from a line passing between the radiological tears [10] (Figure 1). Incidence of implant complications (dislocations and infections) were obtained from medical records.

**Figure 2:**
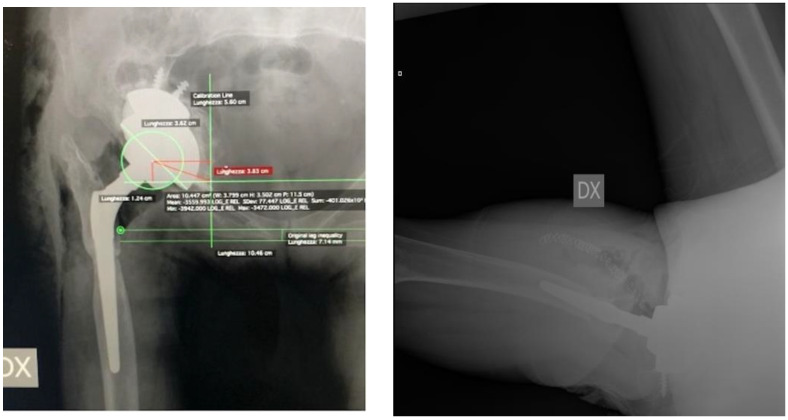
Post-operative radiograph (AP view and axial projection) shows AP pelvis and axial femoral view of the same patient presented in Figure 1 who sustained acetabular revision arthroplasty with revision cup and augment. The two lines positioned at the tip of the lesser trochanter of each femur, respectively, were used to measure the limb length discrepancy. In the present case, the post-operative limb length discrepancy was 7.14 mm.

Radiolucent lines adjacent to the acetabular implant, including any augments, according to DeLee and Charnley [17,18], indicating mechanical loosening of the components, were evaluated on the post-operative radiographs. The cup was considered unstable if a radiolucent line with at least 1 mm in width crossed all three acetabular zones—upper third, medial, or lower third—according to DeLee and Charnley, or if a migration of components could be observed (Figure 2). The fibrous stability of the cup was characterized by a radiolucent line less than 1 mm in width crossing two of the three acetabular zones. The whole construct was considered stable in the presence of bone growth and if the prosthetic components were in close contact with the pelvic bone in the absence of radiolucent lines in at least two of the three acetabular areas [19]. Loosening was defined by the presence of a change greater than 10° in the cup abduction angle, or whether the entire cup moved 6 mm or more on the vertical plane, or on the horizontal plane from its post-operative position [20]. The presence of heterotopic ossification was evaluated according to the Brooker classification [21].

### 2.2. Design of 3D Models

**Figure 3:**
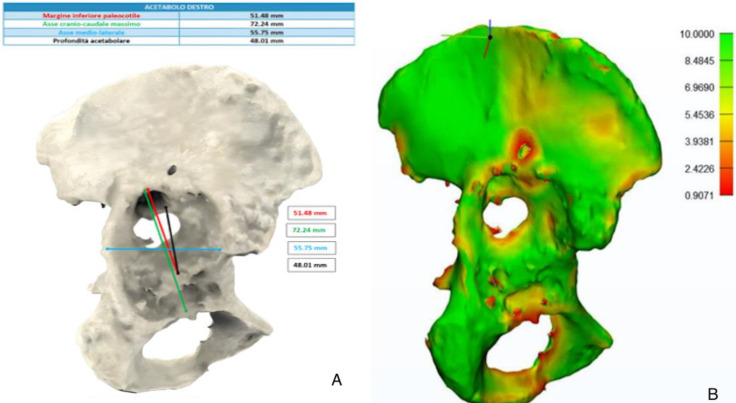
(**A**): Representation of the measurements made on the 3D model; (**B**): bone thickness indicated by color variations on the various areas of the pelvis.

**Figure 4:**
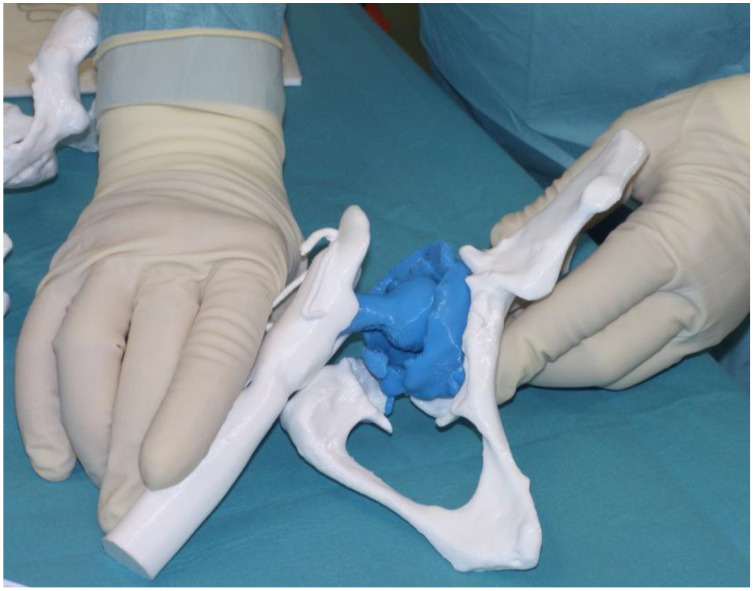
Intraoperative view of the sterilized 3D-printed model of the patient shown in Figure 1 and Figure 2; the blue color indicates acetabular and femoral prosthetic components, while the white color indicates pelvic bone and femoral bone.

Three-dimensional-printed models and a patient-specific anatomical reconstruction model hyper accuracy 3D (HA3D™) were developed for all patients enrolled, and the reconstructions were provided by an independent company (Medics Srl, Turin, Italy) with high expertise in the field. A bilateral CT scan with a metal artifact removal algorithm including the iliac wing and half of the femur or 3 cm below the femoral stem at least was required. The DICOM files were transferred to the company through a cloud-based portal (MyMedics Portal, Turin, Italy). Three-dimensional-printed models were created using HA3D™ reconstruction technology with the ultimaker S5 Pro-bundle model 3D printer; it was able to support all types of filament and offered a simple and intuitive solution for the development of the 3D prototypes. Polylactic (PLA) filament was adopted for all case, as it is an eco-friendly material perfect for fused deposition modeling (FDM) 3D printing, which is a printing technology that allows the production of models with a structure composed of an external rigid shell and a reticulated internal structure—an element that faithfully reproduces the real bony tissue in both the cortical and trabecular parts. Standard measurements and angles were calculated as a second step, and the residual pelvic and acetabular bone stock was displayed as a color map on a dedicated section provided by the portal, allowing the correct visualization of the HA3D^®^ reconstruction and the case analysis. A 3D digital model of the pelvis and lower limbs provided can be rotated by any angle and axially, and a certain amount of bone tissue can be eliminated by the image processing function separating the different anatomical structures observing the relationship between the acetabulum and the femoral head, the anatomical characteristics of the nerve bundles, and the presence of any implants from multiple angles and directions. Bone thickness was defined on the basis of chromatic variations in a color scale ranging from red (<1 mm) to green (>10 mm); the presence of bone gaps, the presence of osteophytes, and the size and orientation of the implants to be revised were evaluated on the digital model (Figure 3). Life-size 3D-printed models were used in the pre-operative phase to achieve a faithful assessment of acetabular bone defects and during the pre-operative planning, furthermore they will be sterilized and used as patient-specific instruments (Figure 4). The cost of each 3D life-sized printed model was around EUR 1500 per case. Time of fabrication included the 3D models’ segmentation step, design, and 3D printing; they take about 7 days to be finished. For the sterilization, a Sterrad method was used, and each model underwent a 47-min cycle of sterilization.

### 2.3. Statistical Analysis

The analyses of all the patients were performed using SPSS for Mac (version 16.0, SPSS Inc., Chicago, IL, USA). The categorical data collected were gender, pre-operative diagnosis, type of acetabular bone defect, post-operative patient satisfaction, radiological loosening of the acetabular components, radiolucent lines adjacent to the acetabular implant, and/or heterotopic augmentation and ossification. The numerical data collected for the study were age, BMI, HHS values, hip ROM, LLD, and cranial and horizontal migration of the COR. The descriptive statistic was calculated. Statistical significance of improvement in HHS, hip ROM, and LLD values was assessed using the Wilcoxon Two-Tailed Signed Rank Test for the paired sample because the data were not normally distributed. A *p* value of less than 0.05 was considered significant.

## 3. Results

### 3.1. Demographics

In the study period, a total of 303 r-THA were performed in our institution. A total of 278 were excluded because they did not meet the inclusion criteria. The study group included 25 patients (25 hips)—20 (80%) were females, the mean age was 62.9 ± 10.8 years (18–83), and the mean BMI was 26.3 ± 5.3 kg. The diagnosis was aseptic loosening in 24 (96.0%) patients and septic loosening in 1 (4.0%) patient. A total of 16 (64.0%) patients underwent cup revision alone. A total of 9 (36.0%) underwent total hip arthroplasty revision of both the cup and the stem. The number hip of previous surgeries was 1 in 12 (48.0%) patients, 2 in 6 (24.0%) patients, 3 in 4 (16.0%) patients, and 4 in 3 (12.0%) patients. A modular multi-hole tantalum cup (Zimmer, Warsaw, Indiana) was used in all patients, with a median diameter of 58.8 (range, 52–66). A Trabecular Metal (TM) augment (Zimmer, Warsaw, Indiana) was added in 8 patients (32.0%), and fixed to the pelvic bone with a median number of 3 screws (range, 2–6). Vitamin E-infused polyethylene insert highwall (E-Poly, Biomet) was cemented in the cup in 14 patients (56.0%), and a dual mobility metal cup that is intended for insertion with cement (Avantage, Zimmer) was implanted in 10 (40.0%) patients. Constrained liner (Zimmer, Warsaw, Indiana) was required in 1 (4.0%) patient. The mean time of surgery was 204.5 ± 47.1 min, blood loss was 463.0 ± 176.9 mL, and LOS was 3.7 ± 0.6 days in the surgery unit and 13.6 ± 6.5 days in the dedicated rehabilitation therapy department. Implant characteristics are summarized in Table 1.

### 3.2. Clinical and Radiographic Results

The mean pre-operative HHS was 35.9 ± 8.4 points, and the post-operative HHS at the final follow-up was 81.2 ± 13.2 points (*p* < 0.001). The mean VAS decreased from 6.7 ± 1.4 points before surgery to 2.4 ± 1.0 points at final follow-up (*p* < 0.001). The number of patients with lameness decreased from 25 cases to 4 cases post-operative: at the time of the last follow-up, 0 (0.0%) patients reported severe or moderate limping, 4 (16.0%) patients reported low limping, and 21 (84.0%) patients reported no limping. The mean LLD of the operated lower limb was 2.0 ± 1.2 cm of shortening before surgery and 0.57 ± 0.6 cm of shortening post-operatively (*p* < 0.001). The mean vertical position of the center of rotation from the line joining the radiological tears changed from 3.5 ± 1.7 cm before surgery to 2.0 ± 0.7 cm after surgery (*p* < 0.05). The mean horizontal position of the center changed from 3.9 ± 1.5 cm before surgery to 3.2 ± 0.5 cm after surgery (*p* < 0.05). The mean acetabular component abduction angle changed from 59.7° ± 29.6° to 46.0° ± 3.9 after surgery (*p* < 0.05). Clinical and radiographic results are reported in Table 2.

Heterotopic ossification was found in 5 (20.0%) of the 25 hips. Of these, 2 (8.0%) patients showed a Grade I according to Brooker, 2 (8.0%) a Grade II, and 1 (4.0%) a Grade III. No patient required additional surgery to remove the ossification. A total of 6 (24.0%) patients reported post-operative complications. A total of 4 (16.0%) patients reported prosthetic dislocations: 3 (12.0%) required further reinterventions and 1 (4.0%) underwent closed reduction. A total of 2 (8.0%) patients reported periprosthetic joint infection within one year from the intervention, and they followed the explant procedure by removal of the mobile prosthetic components (cup insert and head) with preservation of the osteo-integrated and/or cemented prosthetic components, and subsequent targeted long-term antibiotic therapy. The average follow-up was 25.8 ± 2.2 (24–54) months.

## 4. Discussion

The main findings of this study were satisfactory post-operative clinical and radiographic outcomes after a complex hip revision arthroplasty based on a 3D life-sized model. A 3D-printed model provided the surgeon with a physical 3D model of the patients’ specific anatomy, which added supplementary interpretable information, helping in the planning of complex procedures rather than conventional reconstructions. The benefits of data visualization in the transverse plane provided by the 3D-printed model during hip surgery are obvious: the implant type and positioning are better suited to the patient’s anatomy, the joint biomechanics are better restored, and the surgeon’s experience has less of an impact [22]. A 3D life-sized printed model allows the surgeon to try to simulate surgery in the pre-operative setting, and improve the visuo-spatial perception of the bone defects and anatomical relationships between bone and prosthetic implants during revision surgery.

In complex revision hip arthroplasties, the presence of acetabular bone loss might change the surgical strategy defined by Paprosky in his world-renowned protocol [9]. Consequently, proper diagnosis and pre-operative planning are essential for successful treatment. CT is currently the gold standard in the evaluation of periprosthetic osteolysis and bone loss [23,24,25]. Modern CT scanners with high resolution acquisition and suppression of metal artifacts are able to provide localization and characterization of the acetabular bone defects [18]. However, the oblique orientation of the acetabulum and the presence of the acetabular cup can reduce the sensitivity of the CT. In this regard, Fehring et al. showed that the oblique CT reconstruction of 45 degrees allows the frontal visualization of the acetabulum, increasing the sensitivity from 73% (with traditional CT reconstructions) to 91% (with reconstruction at 45 degrees) to detect any bone gaps and unrecognized pelvic discontinuities [23]. Using a life-sized 3D-printed model of the hip, the surgeon can try to fit different typologies and sizes of prosthetic component implants into the pre-operative setting. The 3D-printed model offered a faithful representation of the acetabular bone defect subsequently found in the operating room, which made it easier to evaluate than conventional radiographic or TC images. Paprosky himself highlighted in his study that about 10% of his patients did not show signs of pelvic discontinuity on a standard radiographic, but which was later found during surgery [15]. The trial of the prosthetic implant and the 3D life-sized model also allowed us to evaluate the degree of coverage of the cup to favor bone growth, its limiting of the rate of dislocation, and also the orientation of the screws and augments towards the areas with increased bone thickness. In the pre-operative phase, the type and size of trabecular metal (TM) trial cup also allowed us to better evaluate the type of insert, the range of motion, the size of the head, the restraint options, the dual mobility options, and other specific options which could be potentially used for the selected implant. Comparing the pre-operative and post-operative radiographic evaluations, we observed that the hip to be revised had COR in an abnormal position, and generally displaced cranially and laterally. Nevertheless, despite the complexity of the cases, we were able to restore the COR in an anatomical position, improving the function of the pelvic muscles and hip biomechanics, allowing a reduction in limb length discrepancy, lameness, and time to recovery of walking ability, which took place on the same day of the operation, with the help of a walker.

Aprato et al. compared the effectiveness of traditional CT and 3D models for identifying pelvic discontinuity [26]. All patients with clinical confirmation of pelvic discontinuity were identified with both techniques. The most relevant differences found were higher specificity via the 3D model, and higher positive predictive value due to the absence of artifacts caused by the prosthetic implant and also to the possibility of evaluating the acetabulum without the cup. Tu et al. reported the advantages of planning through 3D printing in patients with Crowe IV Congenital Hip Dysplasia. According to the authors, thanks to the improved spatial orientation, the 3D digital anatomical model promotes the surgical process, allowing the surgeon to understand the anatomical structure of the affected hip and its adjacent area prior to surgery [27]. In fact, by simulating the intervention, corrective methods and preventive measures are taken into consideration in advance for possible problems that may be encountered in the operating room, also benefiting the average surgical time. Li et al. reported in their study a reduction in the average of surgical times and blood losses, to the advantage of a desirable reduction in the risk of infective complications and a more rapid recovery in the post-operative period [28]. The reduction in surgical times would also lead to a reduction in management costs for each individual patient. Ballard et al. analyzed several studies in the literature, identifying the cost per minute in the operating room and quantifying the time saved in the operating room using 3D models [29]. According to the author, this method allows an average reduction of 23 min per individual patient and a saving of USD 1388 per case. Three-dimensional-printed anatomical models can be used as teaching tools of orthopedic surgeons during the training phase for revision total hip arthroplasty. Smith et al. evaluated the useful application of 3D models in anatomy education as a teaching tool in their own right, as well as a method for enriching the curriculum and complementing established learning modalities, such as dissection-based teaching [30].

This study has some limitations. Firstly, this study used a single surgeon who was highly experienced in the prosthetic field, therefore, we cannot assume the utility of the 3D model for highly complex hip surgery performed by less experienced surgeons or surgeons with different levels of experience. Secondly, the sample size was relatively small, which limits the generalizability of the findings; these complex cases had low incidence, and data collection lasted only from April 2019 to October 2020. Thirdly, the lack of a control group limits the comparison with a group who underwent revision surgery traditionally, without 3D life-sized printed models. Fourthly, the duration of the follow-up was limited to two years. More interventions and long-term follow-ups will be needed to understand the real benefits of this method. The main strength of this study is that it relies on complex and severe acetabular revision arthroplasties performed by a single senior surgeon in a single, high-volume center, therefore, the centralized patient selection enhances the homogeneity of perioperative treatment, providing a more consistent and specific analysis.

A future research direction is to conduct another study comparing a cohort of patients who underwent complex hip revision arthroplasty based on a 3D life-sized model with a matched cohort of patients who underwent complex hip revision arthroplasty without the 3D life-sized model in order to compare the clinical and radiographic results, duration of surgery, and blood loss.

## 5. Conclusions

The preliminary clinical application of the new 3D-printed model showed satisfactory clinical and radiographic results after a complex hip revision arthroplasty.

Three-dimensional-printed models allow an accurate assessment in the pre-operative and operative phases of the type of defect, and, consequently, are the best surgical strategy. We believe that the benefits brought by this technique have a greater impact compared to its costs, which are, in addition, going to decrease with the spread of this methodology.

However, the efficacy of this method should be compared to the traditional technique with a long-term follow-up.

## Figures and Tables

**Table 1:** Implant characteristics.

Case Number	Shell Diameter (mm)	Augment	Insert	Stem Revision	Bone Graft	Cage	Bioball	Previous Surgeries (n°)
1	60	No	Avantage	No	No	No	No	3
2	52	No	E-Poly	Yes	No	No	No	2
3	56	No	Avantage	Yes	No	No	No	3
4	58	Yes	E-Poly	Yes	No	No	No	4
5	66	Yes	E-Poly	No	Yes	No	No	4
6	56	No	Avantage	Yes	No	No	No	2
7	66	No	Avantage	No	Yes	No	No	2
8	56	Yes	E-Poly	No	Yes	No	Yes	1
9	60	Yes	E-Poly	No	No	No	No	2
10	50	Yes	E-Poly	No	No	No	Yes	2
11	70	No	Avantage	No	No	Yes	Yes	3
12	60	No	E-Poly	Yes	No	Yes	No	1
13	60	Yes	Avantage	No	Yes	No	No	1
14	52	No	Constrained liner	No	No	No	No	1
15	68	No	Avantage	No	Yes	Yes	No	1
16	No	No	E-poly	No	No	Yes	No	1
17	70	Yes	Avantage	Yes	Yes	Yes	No	1
18	58	No	E-Poly	No	No	No	No	1
19	52	Yes	E-Poly	No	No	No	No	1
20	42	No	E-poly	Yes	Yes	No	No	1
21	70	No	Avantage	No	No	Yes	No	1
22	60	No	Avantage	Yes	No	No	No	3
23	56	No	E-poly	No	No	No	No	1
24	52	No	E-poly	Yes	No	No	No	4
25	58	No	E-poly	No	No	No	Yes	2

**Table 2:** Clinical and radiographic results.

	Pre-Operative (N° = 25)	Post-Operative (N° = 25)	*p* Value
HHS	34.8 ± 8.1	81.6 ± 10.4	*p* < 0.001
VAS	6.7 ± 1.4	2.4 ± 1.0	*p* < 0.001
Severe Limping	14	0	
Moderate Limping	7	0	
Low Limping	4	4	
None Limping	0	21	
LLD (cm)	2.0 ± 1.2	0.6 ± 0.6	*p* < 0.001
Average COR vertical position (cm)	3.5 ± 1.7	2.0 ± 0.7	*p* < 0.05
Average COR horizontal position (cm)	3.9 ± 1.5	3.2 ± 0.5	*p* < 0.05
Acetabular component abduction angle	59.7 ± 29.6	46.0 ± 3.9	*p* < 0.05

HHS, Harris hip score; VAS, visual analogue scale; LLD, leg length discrepancy; COR, center of rotation.

## Data Availability

Data generated or analyzed during this study are included in this published article. Supplementary datasets used and/or analyzed during the current study are available from the corresponding author on reasonable request.

## References

[1] Andrzejewski K., Domżalski M., Komorowski P., Poszepczyński J., Rokita B., Elgalal M. (2023). Optimization of Revision Hip Arthroplasty Workflow by Means of Detailed Pre-Surgical Planning Using Computed Tomography Data, Open-Source Software and Three-Dimensional-Printed Models. Diagnostics.

[2] Park J.-W., Won S.-H., Moon S.-Y., Lee Y.-K., Ha Y.-C., Koo K.-H. (2021). Burden and Future Projection of Revision Total Hip Arthroplasty in South Korea. BMC Musculoskelet. Disord..

[3] Karachalios T., Komnos G., Koutalos A. (2018). Total Hip Arthroplasty: Survival and Modes of Failure. EFORT Open Rev..

[4] Bücking T.M., Hill E.R., Robertson J.L., Maneas E., Plumb A.A., Nikitichev D.I. (2017). From Medical Imaging Data to 3D Printed Anatomical Models. PLoS ONE.

[5] Goh G.D., Sing S.L., Lim Y.F., Thong J.L.J., Peh Z.K., Mogali S.R., Yeong W.Y. (2021). Machine Learning for 3D Printed Multi-Materials Tissue-Mimicking Anatomical Models. Mater. Des..

[6] Duan X., Wang B., Yang L., Kadakia A.R. (2021). Applications of 3D Printing Technology in Orthopedic Treatment. BioMed Res. Int..

[7] Segaran N., Saini G., Mayer J.L., Naidu S., Patel I., Alzubaidi S., Oklu R. (2021). Application of 3D Printing in Preoperative Planning. J. Clin. Med..

[8] Zhang H., Guan J., Zhang Z., Chen X., Ma X., Zhao J., Zhou J. (2022). Restoring Rotation Center in Total Hip Arthroplasty for Developmental Dysplasia of the Hip with the Assistance of Three Dimensional Printing Technology: A Pilot Study. Orthop. Surg..

[9] Yu A.W., Duncan J.M., Daurka J.S., Lewis A., Cobb J. (2015). A Feasibility Study into the Use of Three-Dimensional Printer Modelling in Acetabular Fracture Surgery. Adv. Orthop..

[10] Small T., Krebs V., Molloy R., Bryan J., Klika A.K., Barsoum W.K. (2014). Comparison of Acetabular Shell Position Using Patient Specific Instruments vs. Standard Surgical Instruments: A Randomized Clinical Trial. J. Arthroplast..

[11] Liang H., Ji T., Zhang Y., Wang Y., Guo W. (2017). Reconstruction with 3D-Printed Pelvic Endoprostheses after Resection of a Pelvic Tumour. Bone Jt. J..

[12] La Camera F., Loppini M., Della Rocca A., de Matteo V., Grappiolo G. (2020). Total Hip Arthroplasty with a Monoblock Conical Stem in Dysplastic Hips: A 20-Year Follow-Up Study. J. Arthroplast..

[13] Javan R., Ellenbogen A.L., Greek N., Haji-Momenian S. (2019). A Prototype Assembled 3D-Printed Phantom of the Glenohumeral Joint for Fluoroscopic-Guided Shoulder Arthrography. Skeletal Radiol..

[14] Paprosky W.G., Perona P.G., Lawrence J.M. (1994). Acetabular Defect Classification and Surgical Reconstruction in Revision Arthroplasty. A 6-Year Follow-up Evaluation. J. Arthroplast..

[15] Sporer S.M., Paprosky W.G. (2006). Acetabular Revision Using a Trabecular Metal Acetabular Component for Severe Acetabular Bone Loss Associated with a Pelvic Discontinuity. J. Arthroplast..

[16] Ranawat C.S., Dorr L.D., Inglis A.E. (1980). Total Hip Arthroplasty in Protrusio Acetabuli of Rheumatoid Arthritis. J. Bone Joint Surg. Am..

[17] DeLee J.G., Charnley J. (1976). Radiological Demarcation of Cemented Sockets in Total Hip Replacement. Clin. Orthop..

[18] Weeden S.H., Schmidt R.H. (2007). The Use of Tantalum Porous Metal Implants for Paprosky 3A and 3B Defects. J. Arthroplast..

[19] Sporer S.M., Paprosky W.G. (2006). The Use of a Trabecular Metal Acetabular Component and Trabecular Metal Augment for Severe Acetabular Defects. J. Arthroplast..

[20] Brooker A.F., Bowerman J.W., Robinson R.A., Riley L.H. (1973). Ectopic Ossification Following Total Hip Replacement. Incidence and a Method of Classification. J. Bone Joint Surg. Am..

[21] Fehring K.A., Howe B.M., Martin J.R., Taunton M.J., Berry D.J. (2016). Preoperative Evaluation for Pelvic Discontinuity Using a New Reformatted Computed Tomography Scan Protocol. J. Arthroplast..

[22] Berhouet J., Samargandi R. (2024). Emerging Innovations in Preoperative Planning and Motion Analysis in Orthopedic Surgery. Diagnostics.

[23] Berry D.J., Lewallen D.G., Hanssen A.D., Cabanela M.E. (1999). Pelvic Discontinuity in Revision Total Hip Arthroplasty. J. Bone Joint Surg. Am..

[24] Sporer S.M., O’Rourke M., Paprosky W.G. (2005). The Treatment of Pelvic Discontinuity during Acetabular Revision. J. Arthroplast..

[25] Abdelnasser M.K., Klenke F.M., Whitlock P., Khalil A.M., Khalifa Y.E., Ali H.M., Siebenrock K.A. (2015). Management of Pelvic Discontinuity in Revision Total Hip Arthroplasty: A Review of the Literature. Hip Int. J. Clin. Exp. Res. Hip Pathol. Ther..

[26] Aprato A., Olivero M., Iannizzi G., Bistolfi A., Sabatini L., Masse A. (2020). Pelvic Discontinuity in Acetabular Revisions: Does CT Scan Overestimate It? A Comparative Study of Diagnostic Accuracy of 3D-Modeling and Traditional 3D CT Scan. Musculoskelet. Surg..

[27] Tu Q., Ding H.-W., Chen H., Shen J.-J., Miao Q.-J., Liu B., Yu G.-W., Huang X.-H., Zhu C.-R., Tang Y. (2022). Preliminary Application of 3D-Printed Individualised Guiding Templates for Total Hip Arthroplasty in Crowe Type IV Developmental Dysplasia of the Hip. Hip Int. J. Clin. Exp. Res. Hip Pathol. Ther..

[28] Li Q., Chen X., Lin B., Ma Y., Liao J.X., Zheng Q. (2019). Three-Dimensional Technology Assisted Trabecular Metal Cup and Augments Positioning in Revision Total Hip Arthroplasty with Complex Acetabular Defects. J. Orthop. Surg..

[29] Ballard D.H., Mills P., Duszak R., Weisman J.A., Rybicki F.J., Woodard P.K. (2020). Medical 3D Printing Cost-Savings in Orthopedic and Maxillofacial Surgery: Cost Analysis of Operating Room Time Saved with 3D Printed Anatomic Models and Surgical Guides. Acad. Radiol..

[30] Smith C.F., Tollemache N., Covill D., Johnston M. (2018). Take Away Body Parts! An Investigation into the Use of 3D-Printed Anatomical Models in Undergraduate Anatomy Education. Anat. Sci. Educ..

